# Flexible bronchoscopy-related safety in patients with severe ARDS

**DOI:** 10.1186/s13054-018-2069-y

**Published:** 2018-06-21

**Authors:** A. Guillon, M.-A. Nay, T. Kamel

**Affiliations:** 1 CHRU de Tours, Service de Médecine Intensive Réanimation, Tours, France; 2 INSERM, Centre d’Etude des Pathologies Respiratoires, U1100 Tours, France; 3 CHRO d’Orléans, Service de Médecine Intensive Réanimation, Orléans, France

There is a lack of information regarding the safety of flexible bronchoscopy (FB) in patients with severe acute respiratory distress syndrome (ARDS). We read with great interest the letter by Kalchiem-Dekel and colleagues [1] in which they questioned the feasibility of FB in ARDS patients ventilated in the prone position. Based on retrospective ventilator-related parameters and monitoring data in seven patients, they concluded that FB is feasible in ARDS patients ventilated in the prone position, suggesting that it is safe. We would like to thank the authors for underlining this important question, but we believe that essential physiological aspects are missing in this discussion.

The insertion of the bronchoscope through the endotracheal tube (ETT) increases airway resistance by obstructing the ETT, thereby limiting respiratory flows and ultimately increasing inspiratory and expiratory pressures. If the peak inspiratory pressure is greater than the high-limit pressure set on the ventilator, the tidal volume is not fully delivered. This leads to alveolar derecruitment with well-known consequences in ARDS [2]. If the high-pressure limit is increased to maintain ventilation, the tidal volume is fully delivered but the decrease of expiratory flow results in a trapped volume within the lung that induces dynamic inflation secondary to the incomplete emptying of the lungs at the end of expiration. Hyperinflation increases the alveolar pressure, resulting in a high total PEEP and a high Pplat, possibly leading to pneumothorax in pathological lungs. This cause–effect relationship between ETT obstruction by FB and pneumothorax has been demonstrated by ventilated lung modeling in ARDS [3]. Importantly, the ventilator-measured parameters cannot capture this information during the FB procedure for two reasons. First, respiratory hold maneuvers are required to estimate the alveolar pressures delivered by the ventilator, but it seems inappropriate to perform them during FB. Second, the airway pressures measured by the ventilator during regular hold maneuvers hardly reflect the alveolar pressures because the ETT is obstructed by the bronchoscope. The airway pressures should be measured after the obstruction. Using a manometer placed on the suction channel of the bronchoscope could be an alternative solution [4]. In the individual situations reported by the authors, an immediate increase of +7 cmH2O of alveolar pressure is expected when a FB tube is inserted in the ETT with a 1.7 mm difference in diameters [1, 5]. In conclusion, FB induces unnoticed parenchymal overdistention and derecruitment, which may worsen existing injury; the evolution of the oxygenation parameters could be the tip of the iceberg in terms of FB-related safety in severe ARDS.

## Abbreviations

ARDS: Acute respiratory distress syndrome
ETT: Endotracheal tube
FB: Flexible bronchoscopy
