# Supplementary material for: Flexible bronchoscopy-related safety in patients with severe ARDS
Source: Crit Care. 2018 Jun 21;22:166. doi: 10.1186/s13054-018-2069-y (PMC6013910; doi:10.1186/s13054-018-2069-y)
Supplement: Related texts

# Reply: Authors’ response

**Authors:** Or Kalchiem-Dekel, Ashutosh Sachdeva, Jean Jeudy, Edward M. Pickering, Carl B. Shanholtz

We thank Drs. Guillon, Nay, and Kamel for their interest in our Letter to the Editor [1] and their insightful comments on the physiological consequences of ETT obstruction due to insertion of the bronchoscope in patients with ARDS. The authors’ work on the subject has enhanced our understanding on bronchoscopy in ARDS patients [4, 5]. We acknowledge the importance of clearance between bronchoscope and ETT (in our series it was at least 1.7 mm) to minimize resistance, reduction of minute ventilation, and development of excessive end-expiratory pressure and resultant alveolar overdistention. As outlined by Dr. Guillon and colleagues, these can have potential adverse consequences.

Our data are limited by the retrospective nature of the study and the lack of intra-procedural pressure measurements. The primary objective of our case series was to highlight the feasibility and possible benefit of secretion clearance in ARDS patients while in the prone position. We point out that all patients who underwent bronchoscopy while in the prone position in our case series had mild to moderate ARDS by the Berlin definition [6], whereas the lung models used by Dr. Guillon and colleagues were for severe ARDS [5]. Additionally, unlike static experimental models, real-life bronchoscopy is a dynamic process. To reduce the risk of derecruitment or increase in end-expiratory pressure, all bronchoscopy procedures in our series were performed in brief sequential cycles and suctioning was performed in accordance with recommendations made by the American Association for Respiratory Care regarding mechanically ventilated patients [7]. In the report by Nay et al., however, a different methodology was employed [5]. Furthermore, in our series there were no instances of post-procedure pneumothorax, a potential complication of breath stacking. This is likely a result of two factors: (a) the high-pressure limit was not increased; (b) application of suctioning may have ameliorated end-expiratory pressure build-up.

Finally, data regarding optimal performance of bronchoscopy in patients with ARDS is scarce. We read with great interest the report by Nay et al. describing a novel flexible bronchoscope designed specifically for preservation of lung protective ventilation in mechanically ventilated patients [4]. We hope that this innovation, in conjunction with better designed studies, will provide us with information needed to develop best practice guidelines for bronchoscopy procedures in patients with ARDS and possibly decrease risk of procedure-related complications.
